# Supplementary material for: Identification of photoperiod-induced specific miRNAs in the adrenal glands of Sunite sheep (Ovis aries)
Source: Front Vet Sci. 2022 Jul 22;9:888207. doi: 10.3389/fvets.2022.888207 (PMC9354845; doi:10.3389/fvets.2022.888207)
Supplement: Supplementary file 1 [file Data_Sheet_1.ZIP › Supplementary file/miRDeep2 file.zip!/miRDeep2 file/Know_Structure/SP42A2/oar-let-7c.pdf]

miRBase precursor : oar-let-7c
Total read count : 121279
oar-let-7c read count : 121193
remaining reads : 86

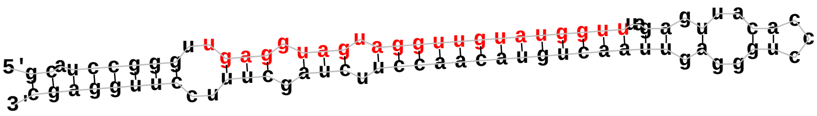

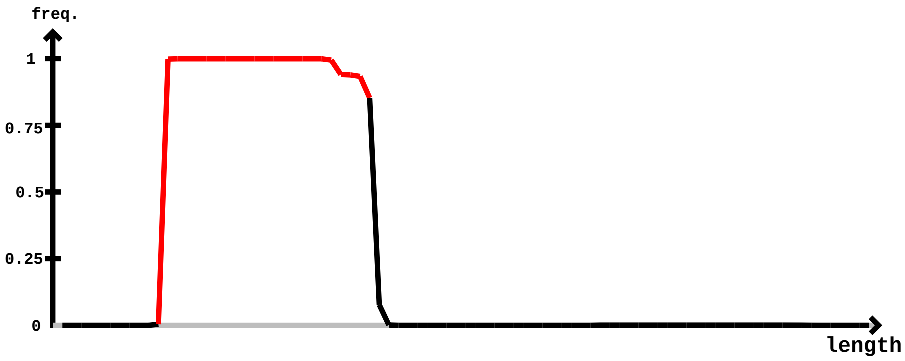


```
               oar-let-7c
5'- gcauccggguugagguaguagguuguaugguuuagaguuacacccugggaguuaacuguacaaccuucuagcuuuccuuggagc   -3'    exp
    ((.((((((..(((.(((.((((((((((((((..((.(..((...))..).)))))))))))))).))).)))..))))))))   reads      mm         sample
    ........guugagguaguagguuguauggu.....................................................   1          0          seq
    ........guugagguaguagguuguaugguu....................................................   1          0          seq
    .........uugagguaguagguuguau........................................................   1          0          seq
    .........uugagguaguagguuguaug.......................................................   2          0          seq
    .........uugagguaguagguuguaugg......................................................   6          0          seq
    .........uugagguaguagguuguauAgu.....................................................   6          1          seq
    .........uugagguaguagguuguauggu.....................................................   155        0          seq
    .........uugagguaguagguuguauAguu....................................................   8          1          seq
    .........uugagguaguagguuguaugguu....................................................   275        0          seq
    .........uugagguaguagguuguaugguuu...................................................   24         0          seq
    ..........ugagguaguagguugua.........................................................   507        0          seq
    ..........ugagguaguagguuUua.........................................................   1          1          seq
    ..........ugagguagGagguugua.........................................................   60         1          seq
    ..........ugaAguaguagguugua.........................................................   6          1          seq
    ..........ugaggCaguagguugua.........................................................   1          1          seq
    ..........ugagguaguaAguugua.........................................................   5          1          seq
    ..........ugagguUguagguugua.........................................................   1          1          seq
    ..........ugagguaguagguuguau........................................................   5887       0          seq
    ..........ugaAguaguagguuguau........................................................   50         1          seq
    ..........ugagguaguaAguuguau........................................................   35         1          seq
    ..........ugagguUguagguuguau........................................................   9          1          seq
    ..........ugagguagGagguuguau........................................................   555        1          seq
    ..........ugagguaguagguuUuau........................................................   15         1          seq
    ..........ugagguaguagguuguaug.......................................................   183        0          seq
    ..........ugagguaguagguuguaugg......................................................   666        0          seq
    ..........ugagguaguagguuguauggu.....................................................   9618       0          seq
    ..........ugagguaguagguuguaugguu....................................................   93656      0          seq
    ..........ugagguaguagguuguaugguuu...................................................   9210       0          seq
    ..........ugagguaguagguuguauAguuua..................................................   68         1          seq
    ..........ugagguaguagguuguaugguuua..................................................   62         0          seq
    ...........gagguaguagguuguau........................................................   3          0          seq
    ...........gagguaguagguuguaugg......................................................   1          0          seq
    ...........gagguaguagguuguauggu.....................................................   7          0          seq
    ...........gagguaguagguuguaugguu....................................................   99         0          seq
    ...........gagguaguagguuguaugguuu...................................................   7          0          seq
```

oar-let-7c

gcauccgggu**ugagguaguagguuguaugguu**uagaguuacacccugggaguuaacuguacaaccuucuagcuuuccuuggagc			
............agguaguagguuguaugguu..	1	0	seq
............agguaguagguuguaugguuu...	1	0	seq
................................uagaguuacacccugggagu................................	1	0	seq
................................uagaguuacacccugggaguu...............................	1	0	seq
................................uagaguuacacccugggaguua..............................	1	0	seq
....aguuaacuguacaaccuucuagcu...........	2	0	seq
....aacuguacaaccuucuagcuu..........	1	0	seq
....aacuguacaaccuucuagcuuu.........	1	0	seq
....cuguacaaccuucuagcuuu.........	5	0	seq
....cuguacaaccuucuagcuuuc........	11	0	seq
....cuguacaaccuucuagcuuucc.......	49	0	seq
....cuguacaaccuucuagcuuuccu......	12	0	seq
....uguacaaccuucuagcuuuccu......	2	0	seq
